# *Carum copticum* L.: A Herbal Medicine with Various Pharmacological Effects

**DOI:** 10.1155/2014/569087

**Published:** 2014-06-25

**Authors:** Mohammad Hossein Boskabady, Saeed Alitaneh, Azam Alavinezhad

**Affiliations:** ^1^Neurogenic Inflammation Research Centre and Department of Physiology, School of Medicine, Mashhad University of Medical Sciences, Mashhad 9177948564, Iran; ^2^Faculty of Agriculture, University of Birjand, Birjand 9719113944, Iran

## Abstract

*Carum copticum* L. commonly known as “Ajwain” is cultivated in many regions of the world including Iran and India, states of Gujarat and Rajasthan. Traditionally, *C. copticum* has been used in the past for various therapeutic effects including bloating, fatigue, diarrhea, abdominal tumors, abdominal pain, respiratory distress, and loss of appetite. It has other health benefits such as antifungal, antioxidant, antibacterial, antiparasitic, and hypolipidemic effects. This plant contains different important components such as carbohydrates, glucosides, saponins and phenolic compounds (carvacrol), volatile oils (thymol), terpiene, paracymene and beta-pinene, protein, fat, fiber, and minerals including calcium, phosphorus, iron, and nicotinic acid (niacin). In the previous studies, several pharmacological effects were shown for *C. copticum*. Therefore, in this paper, the pharmacological effects of the plant were reviewed.

## 1. Introduction

*C. copticum* or Ajwain belongs to the Apiaceae plants family and its seeds are used extensively as a food additive in India and mainly therapeutically effective, with hot nature.* C. copticum *is an Egyptian aborigine plant. This plant grows in arid and semiarid fields in different regions of central Europe, Asia, India (most crops are in the states of Rajasthan, Gujarat, and West Bengal), Iran (especially eastern regions of Baluchistan), Iraq, Afghanistan, and Pakistan [1, 2].

In traditional medicine, different therapeutic applications for* C. copticum* have been described and in Persian traditional medicine it is used for thousands of years [3]. The bronchodilatory, antitussive, and antidyspnea effects were demonstrated for* C. copticum *[3]. The therapeutic effects of this plant in gastrointestinal disorders, such as reflux, cramps, abdominal tumors, abdominal pain, and* Helicobacter pylori*, as well as in eye infection disorders, have been demonstrated [3].

Therapeutic uses of* C. copticum* seeds also include carminative, antiseptic, amoebiasis expectorant, antimicrobial, antiparasitic, antiplatelet-aggregatory, and antilithiasis as well as treating common cold and acute pharyngitis [3]. Abortifacient, galactogogic, and diuretic activities have been observed for this plant [4, 5]. There is also anticarcinogenic potential evidence for* C. copticum* [6]. It has been shown that this plant has also foetotoxicity, abortion potential, and galactogogue properties [7].

In previous studies, different pharmacological effects were shown for* C. copticum*. In addition, the plant has been used widely in traditional medicine. Therefore, different pharmacological effects of* C. copticum* and its constituents were reviewed in the present paper.

## 2. Methods

The following databases and electronic journals were searched from September, 2012, to December, 2013, including Google Scholar, Pubmed, Wiley, Science Direct, and Springer link. Key search terms were* C. copticum*, Ajwain, and* Trachyspermum ammi* and other names of the plant as well as different constituents of the plant and different pharmacological effects. Previously published studies between 1994 and 2014 in the field of different pharmacological effects of* C. copticum* and its different constituents were reviewed in this paper.

## 3. Phytology and Morphology

*C. copticum* is identified in different regions of the world by different names as follows.

Scientific name:* Trachyspermum ammi* and Sprague, it is synonym of* Carum copticum* Benth and in some documents* Aromaticum* has been named by different herbalists.

Different names of the plant in various languages (vernacular name) are Sanskrit: Yamini, Assamese language: Jain, English: Bishop's weed, Hindi, Baluchi: Ajowan and Spirca, Gujarati Language: Ajmo, Canada: Oma, Malaysia: Oman, Arabic: Khella or khellin, Persian: nankhah, zenian, khordaneh, and South Khorasan: ajgho [8].

*C. copticum* is a perennial plant; its height is a little more than black cumin and about a meter, but the leaf shape and color of the flowers of the plant are similar to black cumin. Its stem is ramose; its leaves are slurred and filiform with small white flowers. The plant's fruit which is called* C. copticum* is small, oval, and dark yellow and the fruit surface has five long thin lines of light yellow. Fruits and roots are highly regarded in traditional medicine.

## 4. Chemical Components

The constituents of the seed of* C. copticum* included carbohydrates (38.6%), fat (18.1%), protein (15.4%), fiber (11.9%), tannins, glycosides, moisture (8.9%), saponins, flavone, and mineral matter (7.1%) containing calcium, phosphorous, iron, cobalt, copper, iodine, manganese, thiamine, riboflavin, and nicotinic acid [3, 9].* C. copticum* grows in different areas of the world containing different compounds. Main components of the oil of Iranian and African* C. copticum* oil are carvacrol, *γ*-terpinene, and p-cymene while thymol (97.9%) is the main component of south Indian plant oil. It was also reported that thymol (45.9%), *γ*-terpinene (20.6%), and o-cymene (19%) are the major components of the oil of* C. copticum* but ethylene methacrylate (6.9%), *β*-pinene (1.9%), and hexadecane (1.1%) were the other constituents of the plant [10]. Thymol (72.3%), terpinolene (13.12%), and o-cymene (11.97%) were also identified as constituents of* C. copticum* [11]. Chemical composition of* C. copticum* in two areas in Iran was assessed and results showed that the plant in Kamfiruz contains *γ*-terpinene (48.07%), p-cymene (33.73%), and thymol (17.41%) compared to the composition of plant in Eghlid area which included *γ*-terpinene (50.22%), p-cymene (31.90%), and nerolidol (4.26%) as main components [12].

Chemical constituents of the essential oil of* C. copticum* and its acetone extract were also examined by GC and GC-MS analysis. Results showed that 96.3% of the total amount of the essential oil contains 26 components including thymol (39.1%), p-cymene (30.8%), *γ*-terpinene (23.2%), *β*-pinene (1.7%), and terpinene-4-ol (0.8%) while 68.8% of the total amount of its acetone extract has thymol (39.1%), oleic acid (10.4%), linoleic acid (9.6%), *γ*-terpinene (2.6%), p-cymene (1.6%), palmitic acid (1.6%), and xylene (0.1%) [13]. Hydrodistillation and supercritical fluid (CO2) extraction (SFE) methods of the plant were also performed. In hydrodistilled oil, there were 8 components including thymol (49.0%), *γ*-terpinene (30.8%), p-cymene (15.7%), b-pinene (2.1%), myrcene (0.8%), and limonene (0.7%), but in SFE method with the best condition of temperature, pressure, and dynamic extraction time there were 3 components including *γ*-terpinene (14.2%), p-cymene (23.1%), and thymol (62.0%) [14].

According to the results of study of Srivastava et al., the main constituents of fruit oil of* C. copticum* were p-cymene (41.98%), carvacrol (45.20%), and thymol (0.48%) [15]. The content of chromone, an isomer of the coumarin which is a drug with anticoagulant performance, in various stages of growth of* C. copticum* was determined by high performance liquid chromatography (HPLC) and the results showed that the amount of chromone was higher in unripe than dried [16].

Chemical compounds of* C. copticum* seeds, cultivated in different studies using gas chromatography (GC) and gas chromatography mass spectrometry (GC-MS), are listed in Table 1.

## 5. Pharmacological Effects

*C. copticum* has aromatic odor and spicy taste and is widely used as a spice in the curry powder (curry). The odor of the plant is due to thymol and its aromatic compounds are mainly obtained from methanol extract [19]. Several therapeutic effects were shown for* C. copticum* and its main constituents which were reviewed in the rest of this paper.

### 5.1. Respiratory Effects

One of the therapeutic effects of* C. copticum *is its effect on respiratory system. This plant is used as antiasthma and antidyspnea in traditional medicine. In this context, multiple studies have been carried out including relaxant and inhibitory effects on histamine receptors, stimulatory effect on adrenoreceptors of guinea pigs' tracheal smooth muscles, antitussive effect in guinea pigs, and its bronchodilatory effect on airways of asthmatic patients.

*C. copticum* showed potent relaxant effect on tracheal smooth muscles which was not due to its content of thymol or competitive antagonistic effect on cholinergic receptors. The existence of *α*-pinene in essential oil of this plant showed anticholinergic activity (functional antagonism) [20]. Relaxant effects of different fractions from* C. copticum* including fractions 1, 2, 3, and 4 in guinea pigs' tracheal smooth muscle were shown. For preparation of four fractions, the essential oil was freezed at 0°C overnight. The white crystals were collected by filtration, air dried, and subjected to NMR analysis. The filtrate (1 mL) was chromatographed on a silica gel (70–230 mesh). The column was eluted with solvent mixtures comprising petroleum ether (40–60°C) and chloroform with varying concentrations. Fractions (25 mL) were collected and analogous fractions according to their TLC profile were mixed (solvent system comprising petroleum ether 40–60°C): chloroform (4 : 1) and the spots were visualized using sulfuric acid (50%, v/v). The relaxant effect of fraction 2 of the plant (suggested to be carvacrol) was comparable to the effect of theophylline and more potent than other fractions. Fraction 3 also showed a relaxant effect on tracheal smooth muscle to lesser extent. In addition the results showed that the relaxant effect of fractions 2 and 3 was not due to their inhibitory effect on muscarinic or stimulatory property on beta-adrenergic receptors [21].

Inhibitory effect of* C. copticum* on histamine (H1) receptors of isolated guinea pig tracheal smooth muscle showed a competitive antagonistic effect of the plant on H1 receptors; however, its effect was lower than chlorpheniramine [22].

Stimulatory effect of essential oil, aqueous, and ethanolic extract of* C. copticum* on beta 2 adrenoceptors was examined in isolated guinea pigs tracheal chain. The results showed a stimulatory effect only for ethanolic extract of* C. copticum* on beta 2 adrenoceptors [23]. A xanthine-like activity was also shown for the extract of* C. copticum *[24].

In the study of Gilani et al. bronchodilator effect of* C. copticum* seed extract in presence of high K+ (50 mM) and carbachol on guinea pig tracheal preparation was evaluated. Results demonstrated that* C. copticum *made dose-dependent relaxation (dose 0.1–1 mg/mL) with a possible mechanism of calcium channel blocking effect [25].

The antitussive effects of aerosols of two different concentrations of aqueous and macerated extracts, carvacrol, codeine, and saline were examined by enumerating the number of coughs due to citric acid aerosol 10 min after exposing animals to aerosols of different solutions. Results showed that antitussive effects of aqueous and macerated extracts were similar to codeine which is possibly due to its bronchodilator properties. Nevertheless, carvacrol, one constituent of* C. copticum* with potent bronchodilatory effect, did not show any antitussive effect which suggested different afferent neural route between cough and bronchoconstriction [26].

Bronchodilatory effect of oral administration of boiled extract from* C. copticum* and theophylline in asthmatic patients was also examined. Different pulmonary function tests (FEV1, PEF MMEF, MEF75, MEF50, MEF25, and sGaw) were measured 15 min after administration of different drugs and continued until 180 min after drug administration. The results showed that* C. copticum *has a relatively bronchodilatory effect on asthmatic airways which was comparable with the effect of theophylline at concentrations used [27]. The results of this study suggest that this plant could be of therapeutic value as a bronchodilatory drug in patients with obstructive airway diseases.

One of the main components of* C. copticum* is thymol. The effect of thymol on tracheal and ileum smooth muscles and ciliary motion of respiratory system in rat showed that thymol has a dose-dependent antispasmodic property and increases mucosa transfer due to ciliary motion [28]. Additionally, the antispasmodic effect of thyme extract was demonstrated which is suggested to be due to phenolic volatile oil compounds such as thymol [29].

The relaxant effect of carvacrol, one of the main constituents of* C. copticum,* on tracheal smooth muscle of guinea pigs has been shown which was greater than the effect of theophylline [30].

Other plants containing carvacrol such as* Carum carvi* [31] also showed relaxant effects on tracheal smooth muscle. Fraction 2 of* C. copticum,* which is suggested to be carvacrol, also revealed relaxant effect on tracheal smooth muscle [21]. Therefore, the main constituent of* C. copticum*, carvacrol, may have relaxant effects on the tracheal smooth muscle.

To examine the possible mechanism(s) responsible for the relaxant effect of carvacrol on tracheal smooth muscle, its effect on histamine receptors was evaluated in tracheal smooth muscle of guinea pigs by measuring EC_50_ histamine (effective concentration of histamine causing 50% of maximum response) in the presence of carvacrol and chlorpheniramine. The results of this study showed a competitive antagonistic effect of carvacrol at histamine H1 receptors. In addition, the results suggested its stimulatory effect on *β*-adrenergic receptors and also a blocking effect at muscarinic receptors [32] for carvacrol. In fact, stimulatory effect of carvacrol on *β*2-adrenoceptors was proved by performing isoprenaline concentration response curve and measurement of EC_50_ in the presence of the carvacrol, propranolol, and saline on tracheal smooth muscle of guinea pigs in nonincubated and incubated with chlorpheniramine (to block histamine H1 receptors) conditions. The results showed parallel leftward shift of isoprenaline concentration response curve and lower EC_50_ in the presence of carvacrol and higher EC_50_ in the presence of propranolol compared to the results of saline [33]. These results showed a clear *β*2-adrenoceptors stimulatory effect for carvacrol. In addition, the inhibitory effect of carvacrol on muscarinic receptors which is the other possible mechanism for its relaxant effect on the tracheal smooth muscle was also studied. The rightward shift in methacholine-response curves and the increased EC_50_ in the presence of different concentrations of carvacrol compared with saline were seen which showed possible competitive antagonistic effects of carvacrol at muscarinic receptors [34]. These results suggest that the mechanism of relaxant effect of carvacrol similar to plant extract could have inhibitory effects on muscarinic and histamine receptors and stimulatory effect on *β*2-adrenoceptors or combinations of the three mechanisms.

However, carvacrol with a potent relaxant effect on tracheal smooth muscle shows no antitussive effect [26].

With regard to the lung inflammation in different respiratory diseases, mainly asthma, the anti-inflammatory and immunomodulatory effects of carvacrol were also examined in several studies. The effect of carvacrol on cell culture supernatants of macrophages in porcine induced alveolar inflammatory showed inhibitory effect of carvacrol on TNF-*α*, IL-1*β*, and TGF-*β* [35]. Carvacrol also inhibited secretion of TNF-*α* and IL-1*β* in porcine alveolar macrophage [36]. Anti-inflammatory effect of carvacrol was also evaluated by measurement of exudates volume and leukocyte migration in plural cavity due to carrageenan injection to this cavity which showed a preventive effect of carvacrol on exudates volume and leukocyte migration (*in vivo* and* in vitro*) and suggested an inhibitory effect on COX-1 and COX-2 and 5-lipoxygenase [37]. In addition carvacrol also depicted a preventive effect on serum levels of endothelin, total protein, histamine, NO, and total white blood cells, differential white blood cells (WBC) count and tracheal responsiveness in ovalbumin sensitized guinea pigs [38, 39].  Table 2 summarizes respiratory effects of* C. copticum* and its constituents thymol and carvacrol.

### 5.2. Cardiovascular Effect

Due to calcium channel blocking effect,* C. copticum* has remarkable role in heart rate and blood pressure. Thymol also made fall in blood pressure and heart rate [41]. Several cardiovascular effects of* C. copticum* and its constituents were shown. Negative inotropic and chronotropic effects due to administration of 1–10 mg/kg thymol in mice were shown which lead to decrease in blood pressure. It was suggested that this effect of thymol could be due to calcium channel blocking property [25].

Kumar et al. examined the effect of juice of* C. copticum* leaves on isolated frog heart. It had positive ionotropic effect and negative chronotropic effect on cardiac muscle perfused heart [42].

The cholinomimetic effects of aqueous extracts from* C. copticum* seeds on guinea pigs illume were shown [43], which could cause bradycardia. However, this effect of the plant is not supported by the results of more recent studies. In addition, in a pilot clinical trial, the impact of* C. copticum* on syndrome of cardiovascular disease (angina) was reported which showed that this plant can cause vasodilation of coronary arteries and decreased systemic blood pressure [44].

Lipid-lowering effect of* C. copticum* seeds has been studied in rabbit. In these studies, methanolic extract of the plant (2 g/kg) significantly decreased total cholesterol, triglycerides, and LDL-cholesterol (71%, 53%, and 63%, resp.) and increased HDL up to 60% which was comparable to the effect of simvastatin (0.6 mg/kg). It was also suggested that antilipidemic effect of the plant is possibly due to enhanced removal or catabolism of lipoproteins and inhibition of HMG COA reductase [45, 46]. In addition, it was shown that* C. copticum* seed powder was also effective in increasing secretion of lipase and amylase from pancreas gland in rat [47].

Rajput et al. administered extract of Ajwain with dose of 50 mg/kg and warfarin (0.54 mg/kg) orally to rats and measured coagulation parameters (PT and aPTT). On the 14th day, extract significantly increased PT time compared with warfarin but did not have effect on aPTT. They demonstrated its possible effects on the extrinsic pathway [48].

Administration of thymol orally twice daily (14 mg/kg) to high fat diet rats caused decremented effect on body weight gain and serum lipid peroxidation and increased antioxidant levels [49].

### 5.3. Urogenital Effects

In an* in vivo* study, the effect of the extract of* C. copticum* seeds on urinary stone of 350 patients was investigated. According to data of this study, Ca oxalate, Ca oxalate/uric acid, and Ca-oxalate/hydroxyapatite stones were treated by 100%, 53%, and 31.25%, respectively, with the extract [50]. Recently in India an anticalcifying protein from the seeds of* C. copticum* has been extracted and was administered in urolithiatic rat model. This protein inhibited calcium oxalate deposition by adhesion to calcium oxalate and prevented growth of stones* in vitro* and also* in vivo* [51]. However, other observations did not show any effect of this plant on the production of urea in 24 hours. The results showed that traditional use of* C. copticum* in the treatment of kidney stones was not statistically significant in laboratory setting [52].

*C. copticum* was tested for abortion in some states of India in 1987. The result of the study showed that* C. copticum* leads to abortion in 50 cases of 75 pregnant women and possibly has fetotoxicity feature. However, the possibility of congenital defect in this region of India increased during the study period.* C. copticum* dry seed has phytoestrogen content with 473 ppm value that can increase milk production [9].

Mineraloherbal preparation containing seeds of* C. copticum*, leaves of* Cassia angustifolia* (Senna), fruits of* Terminalia chebula* (Himej) and* Embelia ribes* (Vidang), and roots of* Glycyrrhiza glabra* (Jethimadh) was administered to Sprague-Dawley rats (male and female) by oral route. This preparation reduced number of implantations in females who mated with male rats. However, it did not have significant effect on weight of testis, epididymis, and accessory glands, spermatogenesis, and mating rate in male rats [53].

Effect of* C. copticum* oil on ejaculated human spermatozoa also showed that this plant leads to reduction of sperm activity and pregnancy. Ethanolic extract of* C. copticum* fruit with doses of 100, 200, and 400 mg/kg also was given to male Wistar rats for 60 days. This drug decreased testes weight, number of sperms, and sperm motility dose dependently. In addition, increased level of abnormal sperms was also observed [54]. The viability and membrane integrity of human spermatozoa were evaluated in presence of essential oil of* C. copticum*. This oil reduced viability, antioxidant enzyme, catalase, and mitochondrial function. Cholesterol/phospholipid ratio was increased and therefore the ability of spermatozoa for zygote fertilization is decreased [55]. According to the previous studies* C. copticum* could be a suitable male contraceptive.  Table 3 summarizes the urogenital effects of* C. copticum.*

### 5.4. Gastrointestinal Effects

Traditional use of the* C. copticum* seeds in many gastrointestinal diseases, including intestinal disorders, abdominal pain (colic), or diarrhea, is reported [56]. The alcoholic extract of the plant fruit showed significant reduction effect in ulcer index in an animal model of gastric ulcer [57]. In addition, the extract of crushed fruit from* C. copticum* was effective in relieving stomach pain but increased stomach acid secretion.

Aqueous extract of* C. copticum* (125, 250, and 500 mg/kg) treatment for two weeks improved peptic ulcer induced by ibuprofen in rats which was comparable with the effect of omeprazole. It was also suggested that antiulcer effect of this plant is possibly due to its antioxidant effect [58].

*C. copticum* is able to increase the gastric acid secretion time and the amount of gastric acid. In addition, it was shown that the plant can reduce the transit time of food in the digestive system of mice [59]. Inhibitory effect of* C. copticum* on the contractions of the digestive tract smooth muscle, especially the intestines, increased activities of digestive enzymes and bile secretion was reported [60], which support its effect on gastrointestinal tract.

In several studies, hepatic effects of* C. copticum* have been observed. The effect of 125, 250, and 500 mg/kg from* C. copticum* was assessed on peptic ulcer induced by ibuprofen in rat. In addition, the effect of the extract on liver enzymes including aspartate transferase (AST) and alanine transferase [7] in the serum was examined. Both high and low doses of the extract increase liver enzymes. Thus low dose of this plant is recommended for treatment of peptic ulcer and liver disorders [58].

In a study, the hepatoprotective effects of polyherbal formulations (containing several plants such as* C. copticum*) administered twice daily for one week after paracetamol (500 mg/kg) administration were evaluated on day 8. Paracetamol increases liver enzymes but treatment with polyherbal formulations improved the liver enzyme which was suggested to be due to cell membrane stabilization and recovery of hepatic tissue [61].

The effect of* C. copticum* on liver injury induced by CCL4 and lethal dose of paracetamol (1 g/kg) in mice was also examined. Oral administration of* C. copticum* reduced liver enzymes (ALT, ALP, and AST) and improved paracetamol- and CCl4-induced hepatic injuries [25]. On the other hand, carvacrol caused apoptosis and antiproliferation on HepG2 cells of human hepatocellular carcinoma. Carvacrol selectively decreases phosphorylation of ERK1/2 and activated phosphorylation of p38 but did not affect JNK MAPK phosphorylation. A significant reduction effect on Bcl-2 gene expression was also shown 24 h after carvacrol treatment. In addition, carvacrol inhibited DNA synthesis and decreased the number of cancer cells and total protein content [62].

The effect of* C. copticum* on isolated guinea pig ileum showed antispasmodic activity of extract of the plant and suggested that this effect may be due to cholinergic receptors inhibition by* C. copticum* [63].  Table 4 summarizes The effects of C. copticum on gastrointestinal tract.

### 5.5. Antiparasitic Effects

Infection with filarial nematodes makes lymphatic filariasis and synthetic drug not adequately effective in killing these parasites. Therefore, antifilarial effects of medicinal plant, namely, fruit extract of* C. copticum,* were shown* in vitro* and* in vivo*.* C. copticum,* thymol, and carvacrol have macrofilaricidal properties against adult bovine filarial worm* S. digitata in vitro*. In addition, the plant increased mortality and infertility of female worm of human filarial worm* Brugia malayi in vivo* [65]. The effect of* C. copticum* seeds on treatment of leishmaniasis parasitic was also reported. Hydroalcoholic extract of* C. copticum* showed antileishmanial activity with IC_50_ 15.625 *μ*M which was less than IC_50_ for macrophage cell line (43.76 *μ*M) [66].

Anthelmintic effect of* C. copticum *in comparison with levamisole (an anthelminthic and immunomodulator drug) on sheep infected with mixed nematode was also evaluated.* C. copticum* powder dose dependently caused reduction in eggs per gram of feces which was more potent compared with levamisole [67].

*Plasmodium falciparum* is genus of parasitic protozoa. Infection with this genus is known as malaria. Ethyl acetate extract of* C. copticum* seed with values of 25 *μ*g/mL also showed* in vitro* antimalarial activity [68].

Pinewood nematode (PWN) makes pine wilt disease. Nematicidal activity of* C. copticum* oil against* B. xylophilus* was evaluated* in vitro* and mortality of nematodes after 24 h was studied.* C. copticum *and its components killed nematodes and likely are suitable as natural nematicides. It was also shown that thymol and carvacrol have a significant effect on nematodes [69, 70]. Considering that one of the most important worldwide parasitic diseases (especially in dirty and unsanitary areas) is hydatid cysts, it was shown that* C. copticum* play a significant role in the removal of hydatid cysts* in vitro*. In a study,* Protoscoleces* were exposed to essential oil of* C. copticum* (3, 5, and 10 mg/mL) for 10, 20, 30, and 60 min. The results showed that the higher concentration in the least time period of the study killed 100% of hydatid cyst protoscolices which was suggested to be due to its phenol compounds [71].* Coccidian protozoa* such as* Eimeria tenella* live in intestinal tract of animal and cause coccidiosis which in severe cases lead to death. Herbal complex (containing* C. copticum*) with three concentrations (2, 4, and 6 g) was added to water of broiler chickens infected with* Eimeria tenella* and symptoms were compared with amprolium group. This herbal complex in a concentration-dependently manner improved broiler chickens with* Eimeria tenella* [72].

In addition, there are several studies regarding the disinfecting and insecticide effects of* C. copticum* extracts, such as its effects on adult male and female German cockroaches by inhibition of acetylcholine esterase (AChE). In addition,* C. copticum* oil, 0.1 mg/mL, caused 100% larval mortality against* A. aegypti* mosquito larvae. Thus* C. copticum* can be used as botanical insecticides [73, 74]. The effect of thymol vapor on eggs laying of malaria mosquito (*Anopheles stephensi*) was more effective with LD_50_ 1.6-fold than* C. copticum* oil (80.77 versus 48.88 *μ*g/mL) [75].  Table 5 summarizes antiparasitic effects of* C. copticum*.

### 5.6. The Antimicrobial Effects

Essential oil from Iranian* C. copticum* including 72.3% thymol inhibited gram-positive and gram-negative bacteria and viruses in which inhibition rate is associated with thymol content. High dose of thymol inhibits gram-positive more than gram-negative bacteria. It was shown that phenolic compounds interfere with cell membrane, change pH and ions homeostasis, and perhaps in this way act as antimicrobial agents. At all these studies the antimicrobial activity was examined by broth microdilution method [10, 12, 76, 77].

The effect of aqueous extract of* C. copticum* on several strains of bacteria showed antibacterial effect on* Enterococcus faecalis*,* Staphylococcus aureus*,* Escherichia coli*,* P. aeruginosa*,* S. typhimurium*, and* Shigella flexneri* [78]. The effect of* C. copticum* on fifty-five bacterial strains showed antimicrobial activity with minimum inhibitory concentration <2% (v/v) except* Pseudomonas aeruginosa* [79]. It was also shown that ether fraction of* C. copticum* had better antibacterial and antifungal activity against multidrug resistant (MDR) strains of* Candida albicans*,* Candida krusei*,* Candida tropicalis*,* Candida glabrata*,* Escherichia coli,* and reference strains of* Streptococcus mutans* and* Streptococcus bovis* than other fractions [80].

Dysbiosis disease occurs due to microbial imbalance in intestinal flora as* lactobacilli*,* bifidobacteria*, and coliform bacteria which are lower in fecal counts. In this disease, useful bacteria decreased and harmful bacteria increased in intestinal flora which leads to reduction in energy and body weight. It was shown that* C. copticum* can lead to reduction in pathogenic microorganisms such as* Candida albicans*,* Clostridium *spp.,and* Bacteroides fragilis* while having little effect on microflora and therefore could be effective in dysbiosis treatment [81].

The effect of* C. copticum* with thymol chemotype (when main component is thymol in contrast carvacrol chemotype) on bacterial strains (*S. aureus*,* B. cereus*,* L. monocytogenes*,* E. coli* O157:H7, and* S. enteritidis*) was also evaluated. Bacteria were cultured overnight at 37°C, and the essential oil of the plant and antimicrobial standards (chloramphenicol and ascorbic acid) were added. After incubation at 37°C for 22–24 h, the MIC (mg/mL) was calculated and the microorganism growth inhibition was assayed using an ELISA reader. The results of this study showed that the antimicrobial of* C. copticum* was more potent than* B. persicum* and* C. cyminum* [82].

The antimicrobial effects of* C. copticum* as MIC and MBC were shown in Table 6.

The overall results of these studies showed that* C. copticum* essential oil is rich in monoterpene compounds and could be used as a natural antimicrobial agent in the food and pharmaceutical industries.

Regarding ophthalmic disorders and cataract, it was claimed that the herbal ophthalmic drops (Ophthacare), which is a* C. copticum* extract product, treat infection, inflammation, and cataract in an experimental study [83].

*C. copticum* is also able to protect food against microbial invasion* in vitro*. These antimicrobial properties of* C. copticum* are due to its two ingredients, thymol and carvacrol [84]. Thymol has microbial killing property against common resistant microbial pathogens to multiple antibiotics drugs from the third generation. Therefore, it can be named as the fourth generation plant antibiotic [85].

Gilani et al. studied antibacterial effect of* C. copticum* by applying cream containing 5% essential oil of* C. copticum *to healing wound in rabbits in comparison with iodine tincture. Wound contraction on the 15th day in* C. copticum* group was 99.68%, compared with the healing effect of iodine tincture group, 100%, and nontreatment group, 96.57%, which indicates a wound healing effect of* C. copticum* [86].

In a study, bactericidal properties of* C. copticum* were shown on gram-negative* Erwinia carotovora in vitro* which is suggested to be due to its phenolic compounds such as thymol and carvacrol [87].

### 5.7. Antifungal Effects

Antifungal activity of essential oil of* C. copticum* seeds is also documented against toxigenic* Aspergillus* species. The oil of this plant also is able to inhibit the growth of this parasite [88]. In another study,* C. copticum *(900 ppm concentration)showed fungitoxicity activity against* Epidermophyton floccosum, Microsporum canis, *and* Trichophyton mentagrophytes* [89].

Anti-*Candida* activity of 10–55 *μ*L concentrations of* C. copticum* was assessed by agar disc diffusion assay. The concentrations 50–55 *μ*L of* C. copticum* were more effective in inhibition of the growth of* Candida* [90]. The effect of methanolic extract of* C. copticum* on* Saccharomyces cerevisiae*,* Candida albicans, and C. utilis* was studied* in vitro* which has more effect on* Candida albicans* and* C. utilis* [91].

### 5.8. Antitoxic Effects

Aflatoxins are mycotoxins that are produced by* Aspergillus flavus* and* Aspergillus parasiticus*, species of fungi, which infect crops such as corn and rice;* C. copticum* seed extract is shown to have destructive effect on aflatoxin G1 (AFG1) and significantly reduced aflatoxin activities down to 65%. In addition,* C. copticum* seed extract makes significant damage in other types of aflatoxins (AFB1, AFB2, and AFG2). Damage of aflatoxin G1 caused by* C. copticum* extract was more than 98% in 24 hours, and during 6 hours their destruction has been reported to be over than 78% [92]. The effect of* C. copticum* extract as inhibitors of chromium toxicity has also been shown, as* C. copticum* extract can increase cell viability and decrease DNA damage by reduction of caspase-3 and apoptosis, increase of the mitochondrial membrane potential, and reduction in reactive oxygen species [93].

### 5.9. Neural Effects

*C. copticum* has been used in traditional medicine for relieving rheumatic, joint, headache, and neuralgic pain. Dashti-Rahmatabadi et al. demonstrated that analgesic effect of ethanolic extract of* C. copticum* is comparable with morphine and this effect is suggested to be due to its parasympathomimetic through descending pain modulating pathways [94]. Analgesic effect of* C. copticum* essential oil in formalin test was also assessed and pain scores were recorded during one hour (every 5 minutes). Results showed that essential oil affected the late phase of pain by formalin compared to morphine. The mechanism of this effect of the plant was not due to opioid receptors because it was not reversed by naloxone [95]. Study of Ghannadi et al. on morphine withdrawal syndrome in mice showed that* C. copticum* leads to suppression of morphine withdrawal. It was suggested that this effect was modulated via potentiation of GABA neurotransmission and suppression of glutamate receptors and nitric oxide pathway [96].

Antiepileptic and sedative effects of* C. copticum *in PTZ and amygdala kindling models and its depressant effect in open field test in male rats were also demonstrated and suggested to be due to increase in GABAergic neurotransmission in the brain which reduces neural activity [97].

### 5.10. Dose and Administration Rote

Three to six grams of the seed powder with food or by means of other ways can be consumed daily. Although the seeds are small, they should be powdered for more effectiveness. In addition, it may be extracted or boiled and used. Dried extract of* C. copticum* seeds can be consumed up to 125 mg daily. The liquid extract (tincture) can be also consumed up to 6 mL daily.

### 5.11. Conclusion

*C. copticum* or Ajwain belongs to the Apiaceae plants family and its most important constituents are thymol and carvacrol.

*C. copticum* seeds have various important medicinal properties such as antipyretic, antitussive, antispasmodic and cardiovasodilator, respiratory, liver protection, urogenital, gastrointestinal, antiparasitic, antimicrobial, and lipid lowering effects. Therefore this plant could be of therapeutic value in treating of various disorders. Therefore, further clinical studies regarding various effects of* C. copticum *and its main constituents are recommended. If significant clinical results were found, proper industrial drug products need to be prepared for clinical use.

## Figures and Tables

**Table 1:** Chemical composition of *C. copticum *based on geolocation or type of extraction.

Compounds	Reference [10]	Reference [11]	Reference [12]	Reference [13]	Reference [17]	Reference [18]
Thymol	45.9	72.3%	17.41%, 50.22%	39.1%, 39.1%	54.50%	57.18
*p*-Cymene	—	—	33.73%, 31.90%	30.8%, 1.6%	19.38%	22.55
*γ*-Terpinene	20.6	—	48.07%	23.2%, 2.6%	22.96%	13.07
o-Cymene	19.0	11.97%	—	—	—	—
Ethylene methacrylate	6.9		—	—	—	—
Hexadecane	1.1	—	—	—	—	—
*β*-Pinene	1.9	—	—	1.7%	0.78	0.43
*cis*-Limonene oxide	0.7	—	—	—	—	—
*β*-Myrcene	0.7	—	—	—	—	0.34
Myrcene	—	—	—	—	0.48	—
*α*-Terpinene	—	—	—	—	0.29	0.311
*α*-Thujene	0.4	—	—	—	0.30	
Carvacrol	—	—	—	—	0.46%	0.524
*β*-Phellandrene	0.4	—	—	—	—	0.541
*α*-Pinene	0.3	—	—	—	0.10	0.29
Sabinene	—	—	—	—	—	—
Limonene	0.2	—	—	—	—	—
Dodecane	0.2	—	—	—	—	—
4-Terpineol	0.1	—	—	0.8%	0.11	0.155
*β*-Fenchyl alcohol	0.1	—	—	—	—	—
Terpinolene	—	13.12%	—	—	—	0.095
Nerolidol	—	—	4.26%	—	—	—

**Table 2:** Respiratory effects of *C. copticum* and its constituents thymol and carvacrol.

	Effect	Reference
*C. copticum *	Relaxant effect on tracheal smooth muscles	[20, 40]
	Inhibitory effect of *C. copticum* on histamine (H1) receptors	[22]
	Stimulatory effect on beta 2 adrenoceptors	[23]
	Antitussive effects	[26]
	Bronchodilatory effect	[20, 27]

Thymol	Increase of mucosa transfer due to ciliary motion	[28]
	Antispasmodic effect	[29]

Carvacrol	Relaxant effect	[30, 31, 40]
	Competitive antagonistic effect at histamine H1 receptors	[32]
	Stimulatory effect on *β*-adrenergic receptor	[33]
	Blocking effect at muscarinic receptors	[34]
	Inhibitory effect on secretion of TNF-*α* and IL-1*β* in porcine alveolar macrophage	[35, 37]
	Inhibitory effect on COX-1 and COX-2 and 5-lipoxygenase (anti-inflammatory effect)	[37]

**Table 3:** Urogenital effects of *C. copticum*.

	Effect	Reference
*C. copticum *	Inhibition of calcium oxalate deposition	[51]
	Fetotoxicity feature	[9]
	Increase of milk production	[9]
	Reduction of sperm activity and pregnancy	[54]
	Reduction of testes weight	[55]

**Table 4:** The effects of *C. copticum* on gastrointestinal tract.

	Effect	Reference
*C. copticum *	Antiulcer effect	[58]
	Increase of liver enzymes	[58]
	Increasing of time and amount of gastric acid secretion	[64]
	Inhibitory effect on the gastrointestinal contractions	[59, 60]
	Hepatoprotective effects	[25]
	Antispasmodic effects	[63]

Carvacrol	Apoptosis and antiproliferation effect on HepG2 cells of human hepatocellular carcinoma	[62]

**Table 5:** Antiparasitic effects of *C. copticum*.

	Effect	Reference
*C. copticum *	Macrofilaricidal properties (e.g., *S. digitata*), increase of mortality, and infertility of female worm (*Brugia malayi*)	[65]
	Antileishmanial activity	[66]
	Dysbiosis treatment	[81]
	Anthelmintic effect	[61]
	Antimalarial activity	[68]
	Nematicidal activity	[69, 70]
	Killing of hydatid cyst protoscolices	[71]
	Insecticidal activity	[73, 74]

Thymol	Nematicidal activity	[69, 70]
	Larvicidal activity	[75]

Carvacrol	Nematicidal activity	[69, 70]

**Table 6:** The antibacterial activity of *C. copticum* [82].

Species of bacteria	G	MIC (mg/mL)	MBC (mg/mL)
*S. aureus *	+	0.06	0.06
*B. cereus *	+	0.03	0.03
*L. monocytogenes *	+	0.25	0.25
*E. coli* O157:H7	−	0.25	0.5
*S. enteritidis *	−	0.5	1.0

G (+): gram-positive, G (−): gram-negative.

Minimum bactericidal concentration = MIC.

Minimum inhibitory concentration = MBC.

## References

[1] Zargari A (1996). *Herb*.

[2] Zahin M, Ahmad I, Aqil F (2010). Antioxidant and antimutagenic activity of *Carum copticum* fruit extracts. *Toxicology in Vitro*.

[3] Zarshenas MM, Moein M, Samani SM, Petramfar P (2013). An overview on ajwain (*Trachyspermum ammi*) pharmacological effects; modern and traditional. *Journal of Natural Remedies*.

[4] Chauhan B, Kumar G, Ali M (2012). A review on phytochemical constituents and activities of *Trachyspermum ammi* (l.) Sprague fruits. *The American Journal of Pharm Tech Research*.

[5] Ranjan B (2011). Medicinal uses of *Trachyspermum ammi*: a review. *The Pharma Research*.

[6] Lim TK (2013). *Edible Medicinal and Non-Medicinal Plants*.

[7] Jeet K, Devi N, Thakur N, Tomar S, Shalta L, Thakur R (2012). *Trachyspermum ammi* (ajwain): a comprehensive review. *International Research Journal of Pharmacy*.

[8] Brito-Arias M (2007). *Synthesis and Characterization of Glycosides*.

[9] Bairwa R, Sodha RS, Rajawat BS (2012). *Trachyspermum ammi*. *Pharmacognosy Reviews*.

[10] Mahboubi M, Kazempour N (2011). Chemical composition and antimicrobial activity of *Satureja hortensis* and *Trachyspermum copticum* essential oil. *Iranian Journal of Microbiology*.

[11] Kazemi OR, Behravan J, Ramezani M (2011). Chemical composition, antimicrobial activity and antiviral activity of essential oil of *Carum copticum* from Iran. *Avicenna Journal of Phytomedicine*.

[12] Zomorodian K, Moein MR, Rahimi MJ (2011). Possible application and chemical compositions of *Carum copticum* essential oils against food borne and nosocomial pathogens. *Middle-East Journal of Scientific Research*.

[13] Singh G, Maurya S, Catalan C, de Lampasona MP (2004). Chemical constituents, antifungal and antioxidative effects of ajwain essentials oil and its acetone extract. *Journal of Agricultural and Food Chemistry*.

[14] Khajeh M, Yamini Y, Sefidkon F, Bahramifar N (2004). Comparison of essential oil composition of *Carum copticum* obtained by supercritical carbon dioxide extraction and hydrodistillation methods. *Food Chemistry*.

[15] Srivastava M, Baby P, Saxena A (1999). GC-MS investigation and antimicrobial activity of the essential oil of *Carum copticum* Benth & Hook. *Acta Alimentaria*.

[16] Franchi G, Bovalini L, Martelli P, Ferri S, Sbardellati E (1985). High performance liquid chromatography analysis of the furanochromones khellin and visnagin in various organs of *Ammi visnaga* (L.) Lam. at different developmental stages. *Journal of Ethnopharmacology*.

[17] Mohagheghzadeh A, Faridi P, Ghasemi Y (2007). *Carum copticum* Benth. & Hook., essential oil chemotypes. *Food Chemistry*.

[18] Zeinali T, Khanzadi S, Jamshidi A, Azizzadeh M (2012). Growth response and modeling the effects of *Carum copticum* essential oil, pH, inoculum level and temperature on Escherichia coli O157: H7. *African Journal of Microbiology Research*.

[19] Ishikawa T, Sega Y, Kitajima J (2001). Water-soluble constituents of ajowan. *Chemical and Pharmaceutical Bulletin*.

[20] Boskabady MH, Rakhshandah H, Moetamedshariati V (1998). Bronchodilatory and anticholinergic effects of *Carum copticum* on isolated guinea pig tracheal chains. *Medical Journal of the Islamic Republic of Iran*.

[21] Boskabady MH, Ramazani M, Tabei T (2003). Relaxant effects of different fractions of essential oil from *Carum copticum* on guinea pig tracheal chains. *Phytotherapy Research*.

[22] Boskabady MH, Shaikhi J (2000). Inhibitory effect of *Carum copticum* on histamine (H1) receptors of isolated guinea-pig tracheal chains. *Journal of Ethnopharmacology*.

[23] Boskabady MH, Moemeni A (2000). Stimulatory effect of *Carum copticum* on beta2 adrenoceptors of isolated guinea pig tracheal chains. *Medical Journal of the Islamic Republic of Iran*.

[24] Boskabady MH, Krachian MA (2000). Mechanisms of bronchodilatory effect of *Carum copticum* on isolated guinea pig tracheal chains. *Physiology and Pharmacology*.

[25] Gilani AH, Jabeen Q, Ghayur M, Janbaz K, Akhtar M (2005). Studies on the antihypertensive, antispasmodic, bronchodilator and hepatoprotective activities of the *Carum copticum* seed extract. *Journal of Ethnopharmacology*.

[26] Boskabady MH, Jandaghi P, Kiani S, Hasanzadeh L (2005). Antitussive effect of *Carum copticum* in guinea pigs. *Journal of Ethnopharmacology*.

[27] Boskabady MH, Alizadeh M, Jahanbin B (2007). Bronchodilatory effect of *Carum copticum* in airways of asthmatic patients. *Therapie*.

[28] Begrow F, Engelbertz J, Feistel B, Lehnfeld R, Bauer K, Verspohl EJ (2010). Impact of Thymol in thyme extracts on their antispasmodic action and ciliary clearance. *Planta Medica*.

[29] Engelbertz J, Lechtenberg M, Studt L, Hensel A, Verspohl EJ (2012). Bioassay-guided fractionation of a thymol-deprived hydrophilic thyme extract and its antispasmodic effect. *Journal of Ethnopharmacology*.

[30] Boskabady MH, Jandaghi P (2003). Relaxant effects of carvacrol on guinea pig tracheal chains and its possible mechanisms. *Pharmazie*.

[31] Boskabady MH, Talebi M (1999). Bronchodilatory and anticholinergic effects of Nigella sativa on isolated guinea pig tracheal chains. *Medical Journal of the Islamic Republic of Iran*.

[32] Boskabady MH, Tabanfar H, Gholamnezhad Z, Sadeghnia HR (2012). Inhibitory effect of *Zataria multiflora* Boiss and carvacrol on histamine (H1) receptors of guinea-pig tracheal chains. *Fundamental and Clinical Pharmacology*.

[33] Boskabady MH, Kaveh M, Eftekhar N, Nemati A (2011). *Zataria multiflora* Boiss and carvacrol affect *β*
_2_- adrenoceptors of guinea pig trachea. *Evidence-Based Complementary and Alternative Medicine*.

[34] Boskabady MH, Jafari Z, Pouraboli I (2011). The effect of carvacrol on muscarinic receptors of guinea-pig tracheal chains. *Phytotherapy Research*.

[35] Liu Y, Song M, Che TM, Bravo D, Pettigrew JE (2012). Anti-inflammatory effects of several plant extracts on porcine alveolar macrophages in vitro. *Journal of Animal Science*.

[36] Liu Y (2011). *Effects of Plant Extracts on Immune Function and Disease Resistance in Pigs*.

[37] Fachini-Queiroz FC, Kummer R, Estevão-Silva CF (2012). Effects of thymol and carvacrol, constituents of *Thymus vulgaris* L. essential oil, on the inflammatory response. *Evidence-based Complementary and Alternative Medicine*.

[38] Jalali S, Boskabady MH, Haeri-Rohani A, Eidi A (2013). The effect of carvacrol on serum cytokines and endothelin levels of ovalbumin sensitized guinea-pigs. *Iranian Journal of Basic Medical Sciences*.

[39] Boskabady MH, Jalali S (2013). Effect of carvacrol on tracheal responsiveness, inflammatory mediators, total and differential WBC count in blood of sensitized guinea pigs. *Experimental Biology and Medicine*.

[40] Boskabady MH, Ramazani M, Tabei T (2003). Relaxant effects of eifferent fractions of essential oil from *Carum copticum* on guinea pig tracheal chains. *Phytotherapy Research*.

[41] Aftab K, Usmanghani K (1995). Blood pressure lowering action of active principle from *Trachyspermum ammi* (L.) Sprague. *Phytomedicine*.

[42] Kumar P, Prabhakara M, Satyavathi K, Kumar A (2010). Evaluation of cardiac activity of some traditionally used backyard Indian medicinal plants. *Research Journal of Pharmaceutical, Biological and Chemical Sciences*.

[43] Devasankaraiah G, Hanin I, Haranath P, Ramanamurthy P (1974). Cholinomimetic effects of aqueous extracts from *Carum copticum* seeds. *The British Journal of Pharmacology*.

[44] Anrep GV, Barsoum GS, Kenawy M, Misrahy G (1946). *Ammi visnaga* in the treatment of the anginal syndrome. *The British Heart Journal*.

[45] Javed I, Iqbal Z, Rahman Z (2006). Comparative antihyperlipidaemic efficacy of *Trachyspermum ammi* extracts in albino rabbits. *Pakistan Veterinary Journal*.

[46] Javed I, Khan MZ, Muhammad F (2009). Antihyperlipidaemic efficacy of *Trachyspermum ammi* in albino rabbits. *Acta Veterinaria Brno*.

[47] Ramakrishna Rao R, Platel K, Srinivasan K (2003). In vitro influence of spices and spice-active principles on digestive enzymes of rat pancreas and small intestine. *Food/Nahrung*.

[48] Rajput MA, Khan RA, Qazi N, Feroz Z (2012). Effect of methanol extract of ajwain (*Trachyspermum ammi* L) on blood coagulation in rats. *Journal of the Liaquat University of Medical and Health Sciences*.

[49] Haque MR, Ansari SH, Najmi AK, Ahmad MA (2013). Monoterpene phenolic compound thymol prevents high fat diet induced obesity in murine model. *Toxicol Mech Methods*.

[50] Sabar AG (2010). Lithotripsy of different urinary tract stones by using seeds of *Carum copticum*. *Iraqi Journal of Pharmaceutical Sience*.

[51] Kaur T, Bijarnia RK, Singla SK, Tandon C (2009). *In vivo* efficacy of *Trachyspermum ammi* anticalcifying protein in urolithiatic rat model. *Journal of Ethnopharmacology*.

[52] Ahsan SK, Shah AH, Tanira M, Ahmad MS, Tario M, Ageel AM (1990). Studies on some herbal drugs used against kidney stones in Saudi folk medicine. *Fitoterapia*.

[53] Srivastava SR, Kesarwani S, Keshri G, Singh MM (2005). Evaluation of contraceptive activity of a mineralo-herbal preparation in Sprague-Dawley rats. *Contraception*.

[54] Kumar S (2011). Antifertility effect of *Trachyspermum ammi* (Linn) Sprague fruits on male rats. *International Journal of Pharmaceutical & Biological Archive*.

[55] Paul S, Kang SC (2012). Studies on the viability and membrane integrity of human spermatozoa treated with essential oil of *Trachyspermum ammi* (L.) Sprague ex Turrill fruit. *Andrologia*.

[56] Bentely R (1983). *Medicinal Plants*.

[57] Ramaswamy S, Sengottuvelu S, Haja Sherief SH (2010). Gastroprotective activity of ethanolic extract of *Trachyspermum ammi* fruit. *International Journal of Pharma and Bio Sciences*.

[58] Komeili G, Sargazi m, Solouki S, Maleki S, Saeidi Neek F (2012). Effect of hydroalcholic extract of *Carum copticum* seed on the treatment of peptic ulcer induced by ibuprofen in rats. *Quarterly of Horizon of Medical Sciences*.

[59] Platel K, Srinivasan K (2001). Studies on the influence of dietary spices on food transit time in experimental rats. *Nutrition Research*.

[60] Hejazian S, Morowatisharifabad M, Mahdavi S (2007). Relaxant effect of *Carum copticum* on intestinal motility in ileum of rat. *World Journal of Zooogyl*.

[61] Lateef M, Iqbal Z, Rauf U, Jabbar A (2006). Anthelmintic activity of *Carum copticum* seeds against gastro-intestinal nematodes of sheep. *Journal of Animal and Plant Sciences*.

[62] Yin QH, Yan FX, Zu XY (2012). Anti-proliferative and pro-apoptotic effect of carvacrol on human hepatocellular carcinoma cell line HepG-2. *Cytotechnology*.

[63] Saini N, Singh GK, Nagori B (2014). Hysicochemical characterization and spasmolytic activity of essential oil of ajwain (*Trachyspermum ammi* linn.) from rajasthan. *International Journal of Pharmacological Screening Methods*.

[64] Alqasoumi S (2011). Gastric antisecretory and antiulcer effects of ajowan “*Carum copticum*” in rats. *African Journal of Pharmacy and Pharmacology*.

[65] Mathew N, Misra-Bhattacharya S, Perumal V, Muthuswamy K (2008). Antifilarial lead molecules isolated from *Trachyspermum ammi*. *Molecules*.

[66] Manjili HK, Jafari H, Ramazani A, Davoudi N (2012). Anti-leishmanial and toxicity activities of some selected Iranian medicinal plants. *Parasitology Research*.

[67] Ribero D, Nuzzo G, Amisano M (2011). Comparison of the prognostic accuracy of the sixth and seventh editions of the TNM classification for intrahepatic cholangiocarcinoma. *Hepato Pancerato Biliary*.

[68] Kamaraj C, Kaushik NK, Rahuman AA (2012). Antimalarial activities of medicinal plants traditionally used in the villages of Dharmapuri regions of South India. *Journal of Ethnopharmacology*.

[69] Park IK, Kim J, Lee SG, Shin SC (2007). Nematicidal activity of plant essential oils and components from ajowan (*Trachyspermum ammi*), allspice (*Pimenta dioica*) and Litsea (*Litsea cubeba*) essential oils against pine wood nematode (*Bursaphelenchus xylophilus*). *Journal of Nematology*.

[70] Wright DJ (1981). Nematicides: mode of action and new approaches to chemical control. *Plant Parasitic Nematodes*.

[71] Moazeni M, Saharkhiz MJ, Hosseini AA (2012). *In vitro* lethal effect of ajowan (*Trachyspermum ammi* L.) essential oil on hydatid cyst protoscoleces. *Veterinary Parasitology*.

[72] Zaman MA, Iqbal Z, Abbas RZ, Khan MN (2012). Anticoccidial activity of herbal complex in broiler chickens challenged with *Eimeria tenella*. *Parasitology*.

[73] Yeom H-J, Kang JS, Kim G-H, Park I-K (2012). Insecticidal and acetylcholine esterase inhibition activity of apiaceae plant essential oils and their constituents against adults of German cockroach (*Blattella germanica*). *Journal of Agricultural and Food Chemistry*.

[74] Seo SM, Park HM, Park IK (2012). Larvicidal activity of ajowan (*Trachyspermum ammi*) and Peru balsam (*Myroxylon pereira*) oils and blends of their constituents against mosquito, Aedes aegypti, acute toxicity on water flea, Daphnia magna, and aqueous residue. *Journal of Agricultural and Food Chemistry*.

[75] Pandey S, Upadhyay S, Tripathi A (2009). Insecticidal and repellent activities of thymol from the essential oil of *Trachyspermum ammi* (Linn) Sprague seeds against *Anopheles stephensi*. *Parasitology Research*.

[76] Kazemi OR, Behravan J, Ramezani M (2011). Chemical composition, antimicrobial activity and antiviral activity of essential oil of *Carum copticum* from IRAN. *Avicenna Journal of Phytomedicine*.

[77] Dwivedi SN, Mishra RP, Alava S (2012). Phytochemistry, Pharmacological studies and Traditional benefits of *Trachyspermum ammi* (Linn.) Sprague. *International Journal of Pharmacy & Life Sciences*.

[78] Kaur GJ, Arora DS (2009). Antibacterial and phytochemical screening of *Anethum graveolens*, *Foeniculum vulgare* and *Trachyspermum ammi*. *BMC Complementary and Alternative Medicine*.

[79] Mayaud L, Carricajo A, Zhiri A, Aubert G (2008). Comparison of bacteriostatic and bactericidal activity of 13 essential oils against strains with varying sensitivity to antibiotics. *Letters in Applied Microbiology*.

[80] Khan R, Zakir M, Afaq SH, Latif A, Khan AU (2010). Activity of solvent extracts of *Prosopis spicigera*, *Zingiber officinale* and *Trachyspermum ammi* against multidrug resistant bacterial and fungal strains. *Journal of Infection in Developing Countries*.

[81] Myers SR, Hawrelak JA, Cattley T (2009). Essential oils in the treatment of intestinal dysbiosis: a preliminary *in vitro* study. *Alternative Medicine Review*.

[82] Oroojalian F, Kasra-Kermanshahi R, Azizi M, Bassami M (2010). Phytochemical composition of the essential oils from three Apiaceae species and their antibacterial effects on food-borne pathogens. *Food Chemistry*.

[83] Biswas N, Gupta S, Das G (2001). Evaluation of Ophthacare eye drops—a herbal formulation in the management of various ophthalmic disorders. *Phytotherapy Research*.

[84] Saxena A, Vyas K (1986). Antimicrobial activity of seeds of some ethno-medicinal plants. *Journal of Economic and Taxonomic Botany*.

[85] Khanuja SPS, Srivastava S, Shasney AK Formulation comprising thymol useful in the treatment of drug resistant bacterial infections.

[86] Gilani SR, Mahmood Z, Hussain M (2013). Preliminary evaluation of antimicrobial activity of cream formulated with essential oil of *Trachyuspermum ammi*. *Pakistan Journal of Pharmaceutical Sciences*.

[87] Jafarpour M, Golparvar AR, Lotfi A (2013). Antibacterial activity of essential oils from *Thymus vulgaris*, *Trachyspermum ammi* and *Mentha aquatica* against *Erwinia carotovorain vitro*. *Journal of Herbal Drugs*.

[88] Alizadeh A, Zamani E, Sharaifi R, Javan-Nikkhah M, Nazari S (2010). Antifungal activity of some essential oils against toxigenic *Aspergillus species*. *Communications in Agricultural and Applied Biological Sciences*.

[89] Singh S, Dubey P, Tripathi S (1986). Fungitoxic properties of the essential oil of *Trachyspermum ammi* Sprague. *Mycoses*.

[90] Pirbalouti AG, Bahmani M, Avijgan M (2009). Anti-Candida activity of some of the Iranian medicinal plants. *Electronic Journal of Biology*.

[91] Bonjar GS (2004). Anti yeast activity of some plants used in traditional herbal medicine of Iran. *Journal of Biological Sciences*.

[92] Velazhahan R, Vijayanandraj S, Vijayasamundeeswari A (2010). Detoxification of aflatoxins by seed extracts of the medicinal plant, *Trachyspermum ammi* (L.) Sprague ex Turrill—structural analysis and biological toxicity of degradation product of aflatoxin G1. *Food Control*.

[93] Deb DD, Parimala G, Devi SS, Chakrabarti T (2012). Role of *Carum copticum* seeds in modulating chromium-induced toxicity on human bronchial epithelial cells and human peripheral blood lymphocytes. *Experimental and Toxicologic Pathology*.

[94] Dashti-Rahmatabadi MH, Hejazian SH, Morshedi A, Rafati A (2007). The analgesic effect of *Carum copticum* extract and morphine on phasic pain in mice. *Journal of Ethnopharmacology*.

[95] Hejazian SH (2006). Analgesic effect of essential oil (EO) from *Carum copticum* in mice. *World Journal of Medical Science*.

[96] Ghannadi A, Hajhashemi V, Abrishami R (2012). Effects of the persian *Carum copticum* fruit extracts on morphine withdrawal syndrome in mice. *Research in Pharmaceutical Sciences*.

[97] Rezvani ME, Roohbakhsh A, Mosaddegh MH, Esmailidehaj M, Khaloobagheri F, Esmaeili H (2011). Anticonvulsant and depressant effects of aqueous extracts of *Carum copticum* seeds in male rats. *Epilepsy and Behavior*.

